# Mediatory role of the serum mineral level and discharge disability of stroke survivors 

**DOI:** 10.22088/cjim.15.1.14

**Published:** 2024

**Authors:** Soraya Khafri, Alijan Ahmadi Ahangar, Payam Saadat, Shayan Alijanpour, Mansor Babaei, Mohammadali Bayani, Alireza firouzjahi, Farshad Fadaee Jouybari, Sepideh Hosseini Shirvani, Zahra Frajzadeh, Nafisseh Ezamie

**Affiliations:** 1 Department of Biostatistics and Epidemiology, Babol University of Medical Sciences, Babol, Iran; 2 Mobility Impairment Research Center, Health Research Institute, Babol University of Medical Sciences, Babol, Iran; 3 Students Scientific Research Center, School of Nursing and Midwifery, Tehran University of Medical Sciences, Tehran, Iran; 4 Research and Planning Unit, Pre-hospital Emergency Organization and Emergency Medical Service Center, Babol University of Medical Sciences, Babol, Iran.; 5 Department of Internal Medicine, School of Medicine, Babol University of Medical Sciences, Babol, Iran.; 6 Department of Pathology, School of Medicine, Babol University of Medical Sciences; 7 Ayatollah Rouhani Hospital, Babol University of Medical Sciences, Babol, Iran.

**Keywords:** Stroke, Ischemic stroke, Vita D, Calcium, Magnesium

## Abstract

**Background:**

Possible association between minerals contributing and mortality related to stroke were seen, but prospective data on the relation of vitamin D, magnesium and calcium serum levels with stroke were inconsistent. Consideration about the potential health effects of minerals and nutrients, the current study was conducted.

**Methods:**

This analytical cross-sectional study was conducted on 216 stroke survivors who were referred to the Ayatollah Rouhani Hospital of Babol, Iran. Demographic characteristics, clinical variables, and serum mineral levels were completed in the checklist. Admit score and discharge scale of these patients were determined according to the National Institute of Health Stroke Scale. A path model was constructed to explore the interrelationship between variables and to verify the relationship between variables and disability discharges.

**Results:**

Of 216 stroke patients, 185 (85.6%) cases were ischemic. The discharge status of 29 (12.9%) cases were severe or expired. The patients with moderate and severe admit scores, hemorrhagic stroke type, diabetes mellitus, hypertension and live in the village significantly had a poor discharge disability scale (all of p<0.05). Of all direct paths, Mg (β=-2.85), and among indirect paths, calcium(β=-3.59) had the highest effect on the discharge scale. Only mg had affected the discharge scale through direct and indirect (β=-2.45) paths and had the greatest reverse effect on the discharge scale (β=-5.30; totally).

**Conclusion:**

Hypomagnesemia and hypocalcemia play a mediatory role in poor outcomes. Especially, hypomagnesemia was the direct parameter for poor outcomes. The independent role of each mineral in this issue is difficult to define and suggested for future study.

Stroke is the second leading cause of death worldwide (1, 2). The types, distribution, and contribution of stroke risk factors vary across populations (3-6). Various foods and nutrients are associated with the risk of stroke. Some studies have shown a possible association between minerals contributing and mortality related to stroke. The mineral disorder can contribute to accelerated vascular calcification (7). Some studies found that a diet rich in magnesium(Mg) and calcium(Ca) may decrease the risk of stroke (8). 

Magnesium (Mg) deficiency in the serum of stroke patients has been found. It plays an important role in atherosclerotic processes related to stroke. The low serum Mg level promotes the risk of ischemic stroke (9). furthermore, decreasing the extracellular Mg^2+^concentration around isolated cerebral blood vessels induces rapid cerebrovasospasm (10) that leads to a rapid rise of Calcium (Ca) in brain vascular and neuronal cells which contributes to vasospasm (11). 

The Ca is essential for cellular signaling (12). Calcification results can increase systolic blood pressure (SBP) and pulse-wave velocity which can lead to an increased risk of stroke. In addition, low intake and low serum levels of vitamin D (V.D) were associated with an increased risk of atherosclerosis and cerebrovascular disease (13). Also, adequate 25(OH) D3 is associated with improved absorption of essential elements including calcium, and magnesium (7). 

Turetsky et al. found that with 10 ng/mL reduction in 25 (OH) D concentration was associated with a doubling of the risk of a poor outcome after 90 days (14). Although, these significant effects of VitD are not fully understood (15). Zhang et al. showed that the patients with arterial hypertension, the level of VitD was not associated with the severity of stroke on admission and discharge (16). Controversial results have been reported about the association of calcium, magnesium, and other minerals with stroke (17). 

Furthermore, consideration about the potential health effects of nutrients and minerals such as ca and mg, still remained of interest to the World Health Organization (18). With regard to this issue and doubt about the role of minerals in stroke and limited study in this region, this study was conducted. This study aimed to investigate the impact of serum mineral levels on the discharge disability status of Babol stroke patients.

## Methods

**Setting:** This cross-section analytical study was conducted in Ayatollah Rouhani Hospital of Babol which is a referral center for stroke patients in the North of Iran (5). This study was conducted on 261 patients that referred to this hospital with a diagnosis of stroke. To summarize, any patients with the diagnosis of acute stroke with written informed consent enrolled in this study and the exclusion criteria for stroke cases were similar to the previous study (19).

**Neurological Impairment Grading:** The severity of stroke during the admission time was determined based on National Institute of Health Stroke Scale (NIHSS) criteria. The severity of stroke was mild in score ≤ 8, moderate 9 – 15 and ≥ 16 considered as a severe stroke (20). The degree of disability of patients in the early phase of hospitalization at discharge time(up to the end of the first week) was assessed according to the modified ranking scale(mRS) criteria (21). In the current study, discharge scale was divided into two groups (mRS scale ≤ 3 as mild& moderate vs. mRS scale ≥4 (as severe & death) as in the previous study. 

**Variables**: Demographic characteristics, clinical variables, and severity of stroke survivors in the admission and discharge time were recorded. The definition of underlying disease and laboratory tests were based on the Ahmadi Ahangar et al.’s study and completed in the checklist (19).

**Sample collection and laboratory test:** After diagnosis of stroke in the emergency department, the venous blood samples were collected. Mineral serum levels and routine laboratory tests were performed at this center.

Mg: The serum level of Mg was measured by the Pars Azmoon kit according to the protocol. To determine the concentration of Mg at 450 n, the colorimetric technique was used. 1.5 to 2.5mEq/L (1.8 to 3.0 mg/dL) was considered as normal serum concentrations of Mg^2+ ^(22). 

Ca: Serum calcium level was measured using the Pars Azmoon kit (Tehran, Iran), on the Hitachi e902 with photometric method. The range of 8.2 to 10.5mEq/L was considered as the normal serum concentration of ca^2+^ .

Vit D: At the time of admission, to measure vitamin D level by Euroimmun kit (United States) through the ELISA method (Enzyme-Linked Immunosorbent Assay), four mL blood sample was collected. The range of vitamin D categorized into four groups and under 30 ng/ml considered as low serum level.

a. High (upper than 100ng/ml)

b. Normal Range (30-100 ng/ml)

c. Insufficient group (20-30ng/ml)

d. Deficient group (under 20ng/ml)(23).

**Ethical consideration:** The current study was approved by the Ethics Committee of Babol University of Medical Sciences (MUBABOL.HRI.REC.1394.105). Informed consent was obtained from the participants or their accompanying relatives before any interview or neurologic examination.

**Statistical methods:** Descriptive univariate analysis was conducted for patient characteristics. Path analysis was used to obtain estimates of the main path coefficients, it supplies to regress each (endogenous) dependent variable on those variables that directly imping on it. In other words, standardized regression coefficients (path coefficients) are calculated for obtaining the estimates of each identified path. In this method, the overall effect of a variable on another variable is calculated by adding its “direct effect" and "total indirect effects". The RMSEA, goodness of Fit Index (GFI), Normal Fit Index (NFI), and Comparative Fit Index (CFI) are used in the present study to determine the fit of the model. Data were analyzed using SPSS Version 23 and LISEREL 8.5 software. For modeling, the logistic Regression discharge scale, it is divided into two groups (mild & moderate) and (severe & death). 

## Results

Two-hundred-sixteen stroke patients referred to this center between 2015 to 2016 and were studied. The mean age of these patients was 64.38±13.66 years, 120 (55.6%) cases were females and 155 (71.8%) cases were elderly. The 185 (85.6%) cases were ischemic and 110(50.9%) cases of ischemic patients were embolic.

 Hypertension (HTN) was the most background disease with 120 (55.6%) cases. Stroke impairment in admission time was 121 (56%) cases were mild, 89 (41.2%) were moderate and 6 (2.8%) patients were severe. Furthermore, at the discharge, 139 (64.4%) cases were mild, 49 (22.7%) cases were moderate and 28 (12.9%) patients were severe or expired. Subject characteristic in total and both patients with mild, moderate and severe or expired are shown in table (1). 

Univariate analysis showed gender, age, smoking, ischemic heart disease (IHD) and hyperlipidemia (HLP) between subject characteristic which was not statistically different (all of p>0.05) in both discharge groups. Patients with moderate and severe admit score, with hemorrhagic stroke type, HTN, diabetes mellitus (DM) and living in a village significantly had poor discharge disability scale (all of p<0.05). The low mineral serum levels were statistically significant with discharge disability status except for Vit D (see table 1).

**Table 1:** Characteristics of patients in total and in both discharge disability categories

Variables**	**Mild& Moderate**	**Severe& Death**	**Total**	**P- value**
**Gender**				
**Male**	82 (85.4)^*^	14 (14.6)	96 (44.4)	0.52
**Female**	106 (88.3)	14 (11.7)	120 (55.6)	
**Age(years)**				
**<40**	8 (100)	0 (0)	8 (3.7)	
**40-60**	49 (92.5)	4 (7.5)	53 (24.5)	0.17
**>60**	131 (84.5)	24 (15.5)	155 (71.8)	
**Residential status**				
**Village**	103 (82.4)	22 (17.6)	125 (57.9)	0.01
**City**	85 (93.4)	6 (6.6)	91 (42.1)	
**HTN**				
**Yes**	98 (81.7)	22 (18.3)	93 (43.7)	0.006
**No**	88 (94.6)	5 (5.4)	120 (56.3)	
**DM**				
**Yes**	65 (81.3)	15 (18.8)	80 (37.7)	0.04
**No**	120 (90.9)	12 (9.1)	132 (62.3)	
**Smoking**				
**Yes**	51(86.4)	8 (13.6)	59 (29.4)	0.86
**No**	124 (87.3)	18 (12.7)	142 (70.6)	
**HLP**				
**Yes**	78 (92.9)	6 (7.1)	84 (40.8)	0.12
**No**	104 (85.2)	18 (14.8)	122 (59.2)	
**IHD**				
**Yes**	77 (84.6)	14 (15.4)	91 (44.6)	0.22
**No**	102 (90.3)	11 (9.7)	113 (55.4)	
**Stroke type**				
**Ischemic**	167 (90.3)	18 (9.7)	185 (85.6)	0.001
**Hemorrhagic**	21 (67.7)	10 (32.3)	31 (14.4)	
**Admit Scale**				
**Mild& Moderate**	116 (95.9)	5 (4.1)	121 (56)	<0.001
**Severe**	72 (75.8)	23(24.2)	95 (44)	
**Ca**				
**Normal**	104 (95.4)	5 (4.6)	109 (53.2)	<0.001
**Below**	76 (79.2)	20 (20.8)	96 (46.8)	
**Mg**				
**Normal**	113(97.4)	3 (2.6)	116 (57.1)	<0.001
**Below**	62(71.3)	25 (28.6)	87 (47.9)	
**Vit D**				
**Normal**	43 (95.6)	2 (4.4)	45 (20.8)	0.07
**Below**	145 (84.8)	26 (15.2)	171 (79.2)	

* Values in tables were shown as frequency (percentage)

**Abbreviations: HTN: Hypertension, D.M: Diabetes Mellitus, H.L.P: Hyperlipidemia, I.H.D: Ischemic Heart Disease, Ca: Calcium, Mg: Magnesium, Vit D: Vitamin D.

**Figure 1:**
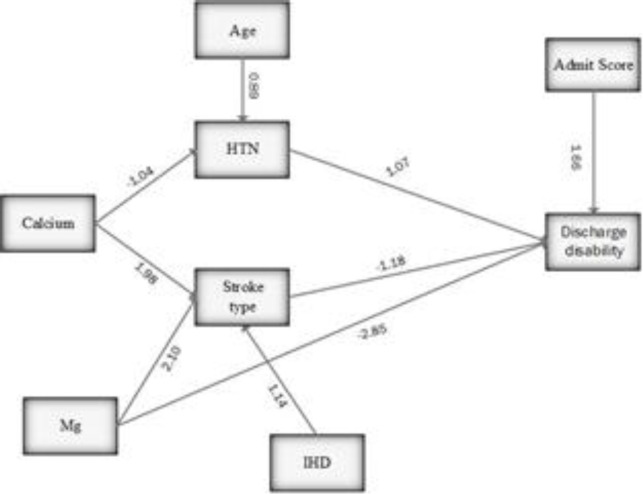
Final path model with standardized path coefficients of predictors of discharge disability. IHD: Ischemic Heart Disease, Vit D: Vitamin D, Mg: Magnesium, Ca: Calcium, HTN: Hypertension

Two model variables of Ca and Mg, which had indirect influences on the discharge scale in two pathways through HTN and stroke type (table 2 and figure 1). Among the two variables, Mg had a direct impact on the discharge scale. The depicted model had the favorable conditions concerning fitting and fitted indices. The goodness of indexes was CMIN/df z=1.63, AGFI=0.97, GFI=0.94 and RMSEA=0.03. Table 2 shows that predictor variable 26% of discharge scale variance. In addition, the models fitted 45% and 38% of HTN and discharge type variances. Table 3 shows standardized coefficients confirming the statistical significance of all indirect and direct effects. According to the results, of all direct paths, Mg (β=-2.85) (and among indirect paths calcium) had the highest effect on the discharge scale. Between all of the variable, only mg had affected discharge scale through both direct and indirect paths and had the greatest reverse effect on the discharge scale (β=-5.3).

**Table 2:** Logistic regression results for predicting the HTN, type of stroke and discharge disability of stroke survivors

**Discharge**	**Type**	**HTN**	Dependents Factors**
1.42 (0.42,4.78)	2.04 (0.61,6.84)	2.36 (1.13,4.94)*	**Age**
0.69 (0.43,2.64)	0.52 (0.147,1.83)	0.98 (0.44,2.19)	**Gender**
0.41 (0.14,1.26)	0.52 (0.176,1.56)	0.89 (0.45,1.76)	**Residential status**
1.17 (0.28,4.86)	2.01 (0.5,8.03)	0.75 (0.3,1.9)	**Smoking**
1.95 (0.69,5.5)	3.52 (1.1,11.23)	0.07 (0.44,1.73)	**IHD**
0.98 (0.16,5.88)	1.31 (0.21,7.97)	0.56 (0.23,1.37)	**Vit D**
13.9 (3.33,57.95)	6.75 (2.09,21.75)	0.59 (0.29,1.19)	**Mg**
2.48 (0.71,8.67)	8.74 (2.2,34.64)	0.39 (0.19,0.76)	**Ca**
2.77 (1.008,8.11)	0.54 (0.16,1.83)	Dependent	**HTN**
0.31(0.11,0.84)	Dependent	-	**Type of stroke**
4.99 (1.73,14.43)	-	-	**Admit score**
Dependent	-	-	**Discharge scale**
0.26	0.38	0.45	**R** ^2^

*Value in table are OR (CI 95%).

**Abbreviations: IHD: Ischemic Heart Disease, Vit D: Vitamin D, Mg: Magnesium, Ca: Calcium, HTN: Hypertension.

**Table 3:** Summary of the direct, indirect and total effects on discharge disability from the path analysis model

**β standardized**			Predictors*
**Total**	**Indirect**	**Direct**	
0.95	0.95	-	**age**
1.07	-	1.07	**HTN**
-3.59	-3.59	-	**Ca**
-5.3	-2.45	-2.85	**Mg**
-1.34	-1.34	-	**IHD**
1.66	-	1.66	**Admit score**
-1.18	-	-1.18	**Type of stroke**

*Abbreviations: HTN: Hypertension, Ca: Calcium, Mg: Magnesium IHD: Ischemic Heart Disease

## Discussion

Due to inconsistence in mineral rols in the stroke and limited related findings in the North of Iran, this study was conducted. A path model was constructed to explore the interrelationship between variables and to verify the relationship between variables and disability discharges. Based on current study findings, difference in mineral serum level except vitamin D had statistically significant association with poor outcome. In current study, hypomagnesemia was directly significant with poor outcome. The significant effect of mg may be due to vasodilator effect. Also, in Beyer’s study, showed that magnesium supplementation may slightly decrease diastolic blood pressure (24). Patients with HTN, DM, rural residency, moderate and severe admit score and hemorrhagic type were significantly had poor outcome or expired. In Ahmadi Ahangar et al.’s study in Babol at 2005, 32.8% of stroke patients were hemorrhagic (25) while in 2016, it decreased to 16 % (5) also, in this study has decreased to 14. The trend of reduction in the prevalence of hemorrhagic type in this area may be due to increasing knowledge of patients in control of HTN or family medicine project in Mazandaran province, North of Iran to follow-up and visit the patients. The third factor of poor early prognosis was DM. While severe discharge status was similar in both diabetic and non-diabetic but more expired cases were seen in diabetic patients. In the Snarska’s study, in-hospital mortality in hemorrhagic stroke was more in DM patients (26), also in the study of Williams, DM was associated with an increased risk of ischemic stroke, and it also changes its clinical picture and worsens the prognosis (27). Both studies presented DM as a risk factor in both stroke types with increasing the mortality rate and worsening the prognosis. The rural residency was statistically significant with poor outcome in the current study. In the study of Mazaheri, found that the stroke prevalences were higher in the urban vs. rural population (28) that was similar with Farghaly et al.’s study (29). Probably, the rural patients may not be inclined or delayed to refer to a hospital that lead to poor outcome.

In the logistic regression models, for HTN between different factors only serum level of Ca and age higher than 60 were the effective factors. Also age upper than 60 years was the parameter for prediction of HTN. 155 (71.8%) cases of stroke survivors were elderly. The difference in age was statistically significant with discharge disability, so severe disability and death was more seen in elderly patients. The 28 (12.9%) patients were severe or death in discharge. Aging is a known risk factor for stroke incidence, it seems that a comprehensive approach and plan should be consider with increasing age.

Between different factors; ischemic type, admit score, serum levels of Mg and HTN were the predictors of the discharge disability status. For ischemic type; IHD, serum level of Mg and Ca were the effective factors. Hypomagnesemia plays a role directly in discharge disability status and indirectly with the effect on ischemic type and finally leads to discharge disability. In discharge period, 28 (12.9%) patients had severe disability or death. 

In Ohira et al.’s study mentioned that HTN and DM could act as mediators between serum magnesium and the incidence of ischemic stroke was more than 95% (30). It seems that serum magnesium was inversely associated with blood pressure and can worsen the prognoses of stroke patients. On other hand, in Leurs et al’s. study, found no result for an overall significant association between calcium concentrations or magnesium with stroke mortality or IHD (18). Also, we should consider other parameters such as serum sodium level, patient care and etc for outcomes of these patients (31, 32). 

This study had several limitations. First, we could not compare the mineral serum level in discharge with admission time. Due to lack of information, we could not assess the diet regims before the stroke. Small sample size and single-center were the limitations of current study. The study had strength points. The results of current study focus on the mediatory role of the serum mineral level with path analysis which limited studies were published with our knowledge. This result presented a new clue in poor outcome of stroke survivors in discharge time. 

The lack of such studies in the North of Iran caused us to conduct this path analysis to assess this issue. Hypomagnesemia, hypocalcemia, ageing and IHD play mediatory role in poor outcome of stroke survivors. On other hand, hypomagnesaemia, admit score, HTN and stroke type were the direct parameters for poor outcome. The independent role of each mineral in this issue is difficult to define. This new clue can be considered in determining the prognosis and in some aspects of therapeutic measures. 
